# P-2193. The Importance of Telemedicine to the Linkage to Care and Treatment of Hepatitis C in West Virginia

**DOI:** 10.1093/ofid/ofae631.2347

**Published:** 2025-01-29

**Authors:** Isabelle Mulango, Nicole Bryan, John A Guilfoose, Kayleigh Burner, Becky Reece, Jesse M Thompson

**Affiliations:** West Virginia School of Medicine, Morgantown, West Virginia; West Virginia University, Morgantown, West Virginia; West Virginia University, Morgantown, West Virginia; West Virginia School of Medicine, Morgantown, West Virginia; West Virginia University, Morgantown, West Virginia; West Virginia University, Morgantown, West Virginia

## Abstract

**Background:**

West Virginia (WV) is entirely located within the Appalachian region and most of the population resides in rural areas. It is currently ranked 2nd in the nation for acute Hepatitis C infections due to the opioid crisis that has devastated the state. The use of direct-acting antivirals (DAA) for Hepatitis C treatment revolutionized care with cure rates near 100%. However, access to treatment remains low, particularly in rural areas such as WV. We implemented telemedicine visits in our Hepatitis C clinic to address this need. Here, we describe the impact of these delivery systems on engagement in care for this population.

**Figure:**
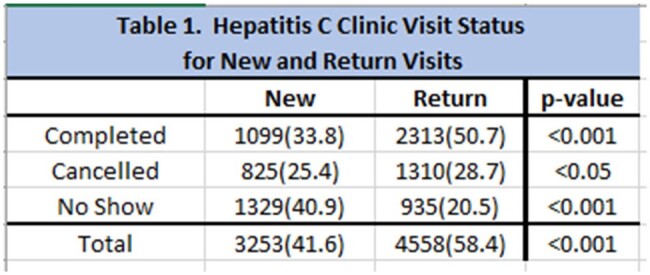

**Methods:**

We conducted a retrospective review of clinic visits at the West Virginia University hospital in Morgantown providing care for individuals with Hepatitis C. Data on adult clinic visits from January 1, 2018 to December 31, 2023 were abstracted from the electronic medical record (EMR) and analyzed by chi-square analysis.

**Figure:**
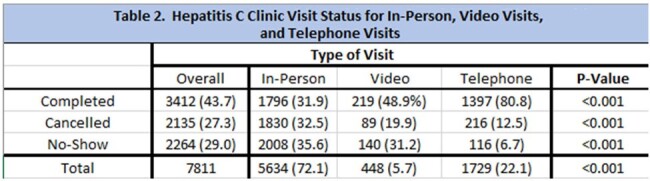

**Results:**

We analyzed a total of 7,811 visits and 2,471 patients. Most of the patients were male (58%, P< 0.001). Ages ranged from 19-87 years old (41 ± 13). The majority of the patients identified as white (92%, P< 0.001), consistent with the racial make-up of the state. 72% of the visits were in-person, compared to 22% telephone and 6% video visits (P< 0.001). 58% of the visits were for return patients vs 42% new visits (P < 0.001). Only 34% of the new patient visits were completed, 25% were cancelled and 41% were no-showed. More telephone visits and video visits were completed (80.8% and 48.9%) than in-person visits (31.9%). The majority of patients were located >25 miles away from the clinic for most visits (66%, P< 0.001). Completion rate for these individuals in-person, telephone and video visits were 32%, 81% and 49%, respectively (P< 0.001).

**Figure:**
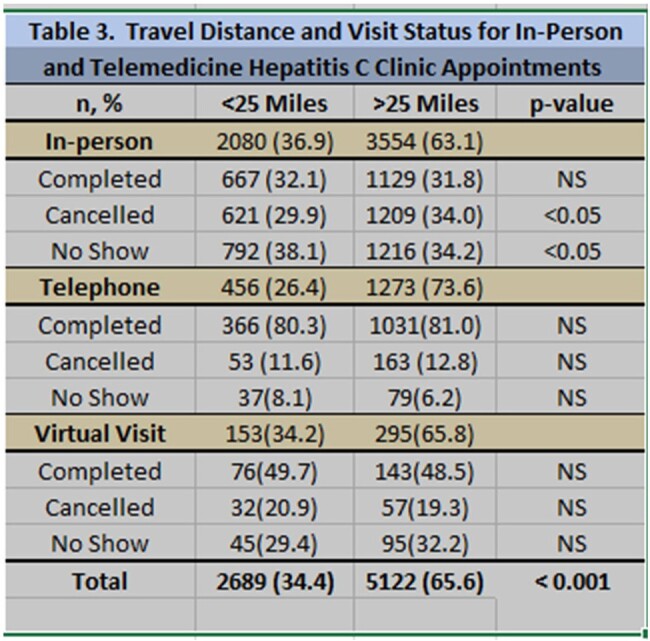

**Conclusion:**

Here, we show that the use of telemedicine was more successful in engaging rural Appalachian patients with Hepatitis C in care compared to in-person visits. Specifically, telephone visits. This finding is likely explained by the limited access to high-speed internet in these areas. Telemedicine services should be increased to expand Hepatitis C care to rural populations.

**Disclosures:**

All Authors: No reported disclosures

